# Alteration of chemokine production in bovine endometrial epithelial and stromal cells under heat stress conditions

**DOI:** 10.14814/phy2.14640

**Published:** 2020-11-23

**Authors:** Shunsuke Sakai, Toshimitsu Hatabu, Yuki Yamamoto, Koji Kimura

**Affiliations:** ^1^ Laboratory of Reproductive Physiology Graduate School of Environmental and Life Science Okayama University Okayama Japan; ^2^ Laboratory of Animal Physiology Graduate School of Environmental and Life Science Okayama University Okayama Japan

**Keywords:** chemokine, cow, endometrial cells, endometritis, heat stress

## Abstract

After parturition, cows frequently develop uterine bacterial infections, resulting in the onset of endometritis. To eliminate the bacteria, bovine endometrial cells secrete chemokines, such as IL‐6 and MCP1, which attract macrophages (MΦs) to the subepithelial stroma. These attracted MΦs are not only involved in bacterial elimination but also the orchestration of inflammation and tissue repair. These immune responses aid in the recovery from endometritis; however, the recovery from endometritis takes longer in summer than in any other season. Based on these findings, we hypothesized that heat stress (HS) affects the chemokine production in endometrial cells. To confirm this hypothesis, we compared IL‐6 and MCP1 production induced by lipopolysaccharide (LPS) in bovine endometrial epithelial and stromal cells under normal (38.5°C) and HS conditions (40.5°C). In the endometrial epithelial cells, IL‐6 production stimulated by LPS was significantly (*p *< .05) suppressed under HS conditions. MCP1 production in endometrial epithelial cells was not detected under both the control and HS conditions regardless of the presence of LPS. Moreover, LPS significantly (*p *< .05) stimulated IL‐6 and MCP1 production in endometrial stromal cells. Moreover, HS significantly (*p *< .05) enhanced their production compared to that under the control conditions. In addition, HS did not affect the migration ability of MΦs; however, the supernatant of the endometrial stromal cells cultured under the HS condition significantly (*p* < .05) attracted the MΦs when compared to the control condition. These results suggest that HS disrupts chemokine production in two types of endometrial cells and alters the distribution of MΦs in the endometrium during the summer.

## Graphical abstract

In present study, the bovine endometrial epithelial and stromal cells were cultured under heat stress condition in the presence of LPS. Heat stress suppressed the inflammatory mediator production in endometrial epithelial cells but enhanced it in endometrial stromal cells.

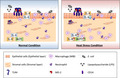

## 1 INTRODUCTION

The bovine endometrium is a major contributor to the regulation of reproduction and is involved in processes such as the estrous cycle, implantation, and placenta formation (Bazer et al., 2009; Poyser, 1995; Roberts et al., 1992). For the endometrium to correctly perform its functions, the uterine lumen is kept aseptic by the cervix and endometrial epithelial cells, which represent anatomical barriers of innate immunity (Sheldon, 2014; Turner et al., 2012). However, because the bovine cervix becomes softer and the endometrial epithelial layer is exfoliated in the postpartum period, the uterine anatomical barrier functions are disrupted, resulting in bacterial infections in the bovine endometrium (Bondurant, 1999; Bromfield et al., 2015). Indeed, in the postpartum period, approximately 20% of cows develop clinical endometritis, which is defined as the presence of a purulent uterine discharge detectable in the vagina (Bromfield et al., 2015). Moreover, 70% of postpartum cows develop subclinical endometritis, which is characterized by the inflammation of the endometrium in the absence of the purulent uterine discharge even after the repair of the endometrium (Sheldon et al., 2008). Because endometritis negatively affects not only uterine function but also ovarian function (Bromfield et al., 2015; Ghanem et al., 2015), recovery from endometritis in cows are necessary before there can be subsequent pregnancies.

The majority of pathogens that cause endometritis are gram‐negative bacteria, such as *Escherichia coli* (Brodzki et al., 2014; Williams et al., 2008). In the bovine endometrium, the response to *E. coli* and its pathogen‐associated molecular patterns, such as lipopolysaccharide (LPS), is dependent on the pattern‐recognition receptors, including the complex of Toll‐like receptor 4 (TLR4), MD‐2, and CD14 (Sheldon et al., 2014). After the detection of LPS by this complex, bovine endometrial epithelial and stromal cells secrete chemokine, such as MCP1, IL‐6, and IL‐8 (Saut et al., 2014; Sheldon et al., 2014) to recruit immune cells to the infection site (Karstrup et al., 2017; MacKintosh et al., 2013). MCP1 and IL‐6 attract macrophages (MΦs) (Bianconi et al., 2018; Clahsen & Schaper, 2008), whereas IL‐8 recruits neutrophils (Caswell et al., 1999). In the case of bovine endometritis, because almost all of *E. coli* are present in the uterine lumen and endometrial epithelial layer, immune cells need to be recruited there to eliminate the bacteria and promote recovery from endometritis (Karstrup et al., 2017). Particularly, the rapid attraction of neutrophils contributes to the elimination of bacteria (Brodzki et al., 2014; Sheldon et al., 2014). In addition, the MΦs are attracted following neutrophils and are not only involved in bacterial elimination but also the secretion of antimicrobial peptides, the orchestration of inflammation, the activation of other immune cells (T‐ and B‐cells) and tissue repair (Dadarwal et al., 2017; Fischer et al., 2010; Garcia et al., 2017; Kauma, 2000; Sheldon et al., 2014).

In the livestock industry, heat stress (HS) is a key concern because it influences not only milk production but also reproductive performance (De Rensis et al., 2015). Specifically, HS leads to the absence of overt estrous behavior because of reduced estradiol 17β production by the dominant follicle and decreases the rate of embryo survival, resulting in a lower conception rate in cows during summer (Cavestany et al., 1985; De Rensis et al., 2015; De Rensis & Scaramuzzi, 2003). Moreover, we previously found that HS directly affects the endometrial cells and disrupts their endocrine function (Sakai et al., 2018). In addition, because it has been reported that cows are at a high risk of endometritis in summer and that its symptoms last longer than in the other seasons (Gautam et al., 2010; Pascottini et al., 2017), we hypothesized that local effect of HS might be involved in not only endocrine function but also the innate immune response in the bovine endometrial cells.

In the present study, to determine whether HS affects the immune responses in bovine endometrium at the cellular level, we examined the chemokine production in bovine endometrial cells in vitro under HS conditions.

## 2 MATERIALS AND METHODS

### 2.1 Collection of uteri

Apparently healthy uteri from cows without a visible conceptus were obtained from a local abattoir (Okayama Meat Center and Tsuyama Meat Center) within 10–20 min of exsanguination and immediately transported to the laboratory, where they were submerged in ice‐cold physiological saline. The stages of the estrous cycle were confirmed by the macroscopic observation of the ovaries as described previously (Ireland et al., 1980).

### 2.2 Isolation and culture of endometrial cells

Uteri at Stage I and Stage IV were used for the isolation and culture of endometrial cells. The epithelial and stromal cells from the bovine endometrium were enzymatically separated and cultured according to procedures described previously (Murakami et al., 2003; Skarzynski et al., 2000). The collected epithelial and stromal cells were separately resuspended in culture medium (DMEM/Ham's F‐12, 1:1 (v/v); Invitrogen) supplemented with 10% (v/v) bovine serum (Invitrogen), 20 µg/ml of gentamicin (Sigma‐Aldrich) and 2 µg/ml of amphotericin B (Sigma‐Aldrich). To culture these epithelial and stromal cells, 1 ml containing 1 × 10^5^ viable cells was seeded in each well of 24‐well dishes (Greiner Bio‐One,) or 75 cm^2^ culture flasks (Greiner Bio‐One), and the cells were cultured at 38.5°C in a humidified atmosphere of 5% CO_2_ in air. When the cells were confluent, the culture medium was replaced with fresh DMEM/Ham's F‐12 supplemented with 0.1% (w/v) BSA, 5 ng/ml of sodium selenite (Sigma‐Aldrich), 0.5 mM ascorbic acid (Wako Pure Chemical Industries), 5 mg/ml of transferrin (Sigma‐Aldrich), 2 mg/ml of insulin (Sigma‐Aldrich) and 20 mg/ml of gentamicin. Then, these cells were used in the following experiments.

### 2.3 Isolation of bovine monocytes

Blood samples were collected from healthy Japanese black cows (*n* = 12)aaa aseptically by venipuncture of the vena jugularis externa into EDTA vacutainer tubes (TERUMO). All procedures were approved by the Animal Care and Use Committee, Okayama University, and were conducted in accordance with the Policy on the Care and Use of the Laboratory Animals, Okayama University (OKU‐2018340). Blood was centrifuged (900 × *g*, 30 min at 25°C) and separated into plasma, buffy coat, and red blood cell sediment. The buffy coat, containing monocytes was resuspended in culture medium (DMEM/Ham's F‐12, 1:1 (v/v)) supplemented with 10% (v/v) bovine serum, 20 µg/ml of gentamicin, and 2 µg/ml of amphotericin B and subsequently seeded in 25 cm^2^ culture flasks (Greiner Bio‐One). After overnight incubation, the culture medium containing unattached cells was removed.

### 2.4 Differentiation of monocytes into macrophage‐like cell (MΦs)

Monocytes were stimulated with 100 ng/ml of bovine INFγ (Gift from Dr. Shigeki Inumaru) for 5 days to induce differentiation into MΦs.

### 2.5 Migration assay

Migration assays were performed in 24‐well plates with ThinCert (Greiner Bio‐One). The upper and lower wells were separated by a polycarbonate membrane (8 μm pore size). The lower wells contained 900 μl of medium, including 300 µl of isotonic Percoll. The upper wells contained MΦs (10 × 10^5^ cell/well), which were stained with Dil (Invitrogen) for 3 hr at 38.5°C. These wells were incubated in a humidified atmosphere of 5% CO_2_ in air. At the end of the incubation, un‐migrated MΦs on the upper ‐side of the polycarbonate membrane was removed from the inserts with a cotton‐tipped swab. The MΦs that migrated to the lower ‐side of the polycarbonate membrane were counted using a fluorescence microscope (OLYMPUS).

### 2.6 Total RNA extraction and quantitative RT‐PCR

Total RNA was extracted from the cultured cells using RNAiso Plus (TaKaRa Bio) according to the manufacturer's directions. The optical density A260/280 of RNA samples was measured by NanoDrop LITE (Thermo Fisher Scientific) and its value was approximately 2. One microgram of each total RNA was reverse transcribed using ReverTra Ace qPCR RT Master Mix with gDNA remover (TOYOBO). Quantification of *MCP1*, *IL6*, *CXCL8*, *CCL3*, *CCL5*, *CX3CL1*, *CXCL10*, *CXCL11*, *TLR4*, *MD2*, *CD14*, *IL6 Receptor*, *CCR2*, and glyceraldehyde‐3‐phosphate dehydrogenase (*GAPDH*) mRNA expression was carried out following a previously described method with some modifications (Sakumoto et al., 2006). Briefly, we quantified the expression of each mRNA using Brilliant III Ultra‐Fast SYBR Green QPCR Master Mix With Low ROX (Agilent Technologies) starting with 2 ng of reverse‐transcribed total RNA. The expression of GAPDH was used as an internal control determined using NormFinder (Andersen et al., 2004). For the quantification of the mRNA expression levels, PCR was performed under the following conditions with the AriaMx Real‐Time PCR System (Agilent Technologies): 95°C for 30 s, followed by 45 cycles of 95°C for 10 s, 56°C or 60°C for 10 s, and 72°C for 15 s with continuous fluorescence measurement. The melting curve was analyzed under the following conditions to check the specificity of the PCR products: 95°C for 1 min, 56°C or 60°C to 95°C with 0.5°C interval, 5 s soak time. Serial dilutions (20–20,000,000 copies) of each PCR product extracted from the agarose gel were used as a standard to analyze each mRNA expression level. The sequence of each primer and the annealing temperatures is listed in Table 1.

**Table 1:** Sequences of primers used for quantitative RT‐PCR analysis

Genes	Forward and reverse primers	Accession no.	Annealing temperature
*MCP1*	5′‐CGCCTGCTGCTATACATTCA‐3′ 5′‐GCTCAAGGCTTTGGAGTTTG‐3′	NM_174006.2	60°C
*IL6*	5′‐AACCACTGCTGGTCTTCTGG‐3′ 5′‐GGTCAGTGTTTGTGGCTGGA‐3′	NM_173923.2	60°C
*CXCL8*	5′‐GGCTGTTGCTCTCTTGGCAG‐3′ 5′‐CTGAATTTTCACAGTGTGGCCC‐3′	NM_173925.2	60°C
*CCL3*	5′‐GCTGACTATTTTGAGACCAG‐3′ 5′‐GGTCGGTGATGTATTCCT‐3′	NM_174511.2	56°C
*CCL5*	5′‐AGGAGTATTTCTACACCAGCAG‐3′ 5′‐GCGTTGATGTACTCTCGCA‐3′	NM_175827.2	60°C
*CX3CL1*	5′‐CAACACGGTGTGAACAAG‐3′ 5′‐CTTGTGGTCTAGGTGCTT‐3′	XM_002694885.5	56°C
*CXCL10*	5′‐CTGCAAGTCAATCCTGC‐3′ 5′‐TGCAGGAGTAGTAGCAGCT‐3′	NM_001046551.2	56°C
*CXCL11*	5′‐CAAGCATGAGTGTGAAGG‐3′ 5′‐CTTTCTCAATATCTGCCAC‐3′	NM_001113173.1	56°C
*TLR4*	5′‐TGCCTGAGAACCGAGAGTTG‐3′ 5′‐ATATGGGGATGTTGTCGGGG‐3′	NM_174198	60°C
*MD2*	5′‐GGGAAGCCGTGGAATACTCTAT‐3′ 5′‐CCCCTGAAGGAGAATTGTATTG‐3′	NM_001046517.1	60°C
*CD14*	5′‐CAGCCGACAACCAGAGAGAG‐3′ 5′‐TAGACCAGTCAGGCTTCGGA‐3′	NM_174008.1	60°C
*IL6 Receptor*	5′‐CATTAGGCGAGGAGGAAGCA‐3′ 5′‐CTGTGGCATTGTCCTCTGGT‐3′	NM_001110785.1	60°C
*CCR2*	5′‐TACTTGCCCTTGTATCTCCG‐3′ 5′‐GTATCATTGCCATCCATCTTC‐3′	XM_015459671.1	60°C
*CD80*	5′‐CTAATCAGCACCTGACCTGG‐3′ 5′‐GTCTGCGTATTGCAGCATTT‐3′	NM_001206439.1	60°C
*GAPDH*	5′‐CTCTCAAGGGCATTCTAGGC‐3′ 5′‐TGACAAAGTGGTCGTTGAGG‐3′	NM_001034034.2	60°C

### 2.7 Enzyme immunoassay (EIA)

The concentrations of MCP1 and IL‐6 in supernatants were measured by EIA using a Bovine MCP1 ELISA Reagent Set (GWB‐KAAO76; GenWay Biotech, Inc) and Bovine IL‐6 ELISA Reagent Kit (ESS0029; Thermo Fisher Scientific), respectively. The concentration of IL‐8 in the culture medium was determined by EIA as described previously (Hirota et al., 2011). The standard curve ranges of MCP1, IL‐6, and IL‐8 were 0.156–10, 0.039–10, and 0.078–20 ng/ml, respectively. The median effective dose (ED_50_) values of the assays for MCP1, IL‐6, and IL‐8 were 1.25, 0.625, and 1.25 ng/ml, respectively. The intra‐ and inter‐assay coefficients of variation, on average, for MCP1, IL‐6, and IL‐8 were 7.5% and 5.8%, 3.52% and 1.12%, 8.39% and 13.52%, respectively.

### 2.8 DNA assay

The DNA content of cultured endometrial cells was measured as described previously (Labarca & Paigen, 1980). Briefly, after cell disruption with an ultrasonic homogenizer, the cell lysates were incubated with Hoechst H 33258 (Sigma‐Aldrich). After incubation for 10 min, the fluorescence of each sample and standard was measured with a microplate fluorometer (Fluoroskan Ascent, Labsystems, Thermo Fisher Scientific). The standard curve ranged from 0.625 to 30 µg/ml, and the ED_50_ of the assay was 10 µg/ml.

### 2.9 Fluorescence immunohistochemistry

The paraffin‐embedded endometrial tissues were sectioned (4 μm), and placed on silane‐coated glass slides (Dako A/S). After deparaffinization with xylene, the sections were subjected to antigen retrieval via heating in 0.01 M citrate buffer (pH 6.0) using a microwave at 600 W for 15 min. The sections were then incubated in 2.5% normal horse serum (Vector Laboratories) at room temperature for 20 min, which was followed by incubation at 4°C overnight with CD172A monoclonal antibody DH59B (BOV2049, Washington State University Monoclonal Antibody Center) diluted in PBS at 1:100. The sections were then treated at room temperature for 1 hr with an appropriate fluorescent second antibody, the Alexa Fluor 488 goat anti‐rabbit IgG antibody (1:500 dilution of A11005; Life Technologies). The sections were washed with PBS and covered with Prolong Gold with DAPI (Life Technologies). The sections were photographed using an FSX100 Bio‐Image Navigator (Olympus).

### 2.10 Experimental design

#### 2.10.1 Experiment 1: Effect of HS and LPS on the cell viability in bovine endometrial epithelial and stromal cells

Bovine endometrial epithelial and stromal cells were cultured under control or HS conditions (Sakai et al., 2018). The control samples were cultured at 38.5°C for 34 hr, followed by the LPS challenge (0 or 1 µg/ml) (LPS from *Escherichia coli* O55:B5; Sigma‐Aldrich, L6529) for 12 hr at 38.5°C. The HS samples were cultured at 40.5°C for 10 hr, allowed to recover at 38.5°C for 14 hr, and then cultured at 40.5°C for 10 hr again, this was followed by the LPS challenge (0 or 1 µg/ml) for 12 hr at 40.5°C. Since 1 µg/ml of LPS significantly upregulates inflammatory mediator mRNA expression and production in bovine endometrial cells (Cronin et al., 2012; Herath et al., 2006) and reflects the concentrations in the uterine lumen of infected cattle (Dohmen et al., 2000; Williams et al., 2007), we performed one dose of the LPS challenge (1 µg/ml) in the present study. After the LPS challenge, the cells were harvested to measure the DNA content.

#### 2.10.2 Experiment 2: Effect of HS and LPS on the mRNA expression of chemokines in cultured bovine endometrial epithelial and stromal cells

Endometrial epithelial and stromal cells were cultured as described in Experiment 1. In this experiment, to measure the gene expression, these cells were exposed to LPS (0 or 1 µg/ml) for 6 hr. We confirmed that the mRNA expression of chemokines was higher at 6 hr compared to 3 and 12 hr (data not shown). After LPS treatment, the cells were harvested to measure the mRNA expression of *IL6, MCP1,* and *CXCL8*, *CCL3*, *CCL5*, *CX3CL1*, *CXCL10*, *CXCL11*. In addition, to confirm whether HS treatment was effictive, we evaluated the heat shock protein 70 and confirmed its increase in both types of endometrial cells (data not shown).

#### 2.10.3 Experiment 3: Effect of HS and LPS on the production of IL‐6, MCP1, and IL‐8 in cultured bovine endometrial epithelial and stromal cells

Endometrial epithelial and stromal cells were cultured as described in Experiment 1. After LPS treatment for 12 hr (Zhang et al., 2019; Zhu et al., 2015), the medium was collected in a 1.5 ml tube and immediately frozen and stored at − 30°C until the measurement of the concentrations of MCP1, IL‐6, and IL‐8 by ELISA. To normalize the concentration of each chemokine, the cells were harvested and measured the DNA content.

#### 2.10.4 Experiment 4: Effect of HS and LPS on the mRNA expression of *TLR4*, *CD14* and *MD2* in cultured bovine endometrial epithelial and stromal cells

Endometrial epithelial and stromal cells were cultured as described in Experiment 2. Since CD14 and MD‐2 are related to the recognition of the LPS in cooperation with TLR4 (Park et al., 2009; Sheldon et al., 2014; Werling & Jungi, 2003), the mRNA expression of *TLR4*, *MD2*, and *CD14* was measured.

#### 2.10.5 Experiment 5: Effect of HS on the mRNA expression of *IL6 Receptor* and *CCR2* in MΦs and their migration ability

First, to confirm the isolation of the monocytes and their differentiation into MΦs, the monocytes were stained using specific and non‐specific esterase (Muto Pure Chemicals Co., LTD), and the morphological change and *CD80* mRNA expression of the differentiated cells were investigated. The differentiated MΦs were incubated at 38.5°C or 40.5°C following a previously described method (Sakai et al., 2018). After incubation, MΦs were harvested to measure the mRNA expression of *IL6 Receptor* and *CCR2*.

To investigate whether HS affects the migration ability of MΦs, migration assays were performed. As control samples, the 24‐well plates with ThinCerts containing the Dil‐stained MΦs in the upper well were cultured at 38.5°C for 34 hr, which was followed by the addition of 50 ng/ml of MCP1 (Bovine MCP1, PBP024, Bio‐Rad Laboratories) in the lower well and further incubation for 12 hr at 38.5°C. As HS samples, the 24‐well plates with ThinCerts that contained the Dil‐stained MΦs in the upper well were cultured at 40.5°C for 10 hr and allowed to recover at 38.5°C for 14 hr; they were then cultured at 40.5°C for 10 hr again, which was followed by the addition of 50 ng/ml of MCP1 in the lower well and further incubation for 12 hr at 40.5°C. After incubation, the migrated MΦs were counted using a fluorescence microscope.

To confirm whether the endometrial stromal cells under the HS condition attracted a large number of MΦs, a migration assay was performed with the culture supernatant of the endometrial stromal cells. The endometrial stromal cells were cultured in 75 cm^2^ culture flasks with 10 ml of BSA‐free culture medium under the same conditions as described in Experiment 3. After this culture medium was concentrated to 600 μl with Centricut (U‐10, cut‐off value of molecular weight: 10,000, Kurabo Industries Ltd.), the migration assay was performed at 38.5°C for 12 hr. Since LPS was also concentrated in this experiment and it might affect the migration ability of the MΦs, the migration assay was performed with the medium which contains the concentrated LPS.

#### 2.10.6 Experiment 6: Localization of MΦs in bovine endometrium in summer and winter

For the measurement of the length of the MΦs from the epithelial layer, endometrial tissues (Stage III) were collected from six cows in the winter (January–February and November–December 2017) and six cows in the summer (July–August 2017). The average temperatures in Okayama during the sampling periods were 6.4°C in the winter and 28.4°C in the summer, respectively. The temperature data were obtained from the Japan Meteorological Agency. The MΦs were stained and 20 positive immunostaining of MΦs were selected randomly. The distance of these MΦs, which are perpendicular to the epithelial layer, was measured via cellSens imaging software (Olympus).

### 2.11 Statistical analysis

All experimental data are shown as the mean ± *SEM*. All the data were tested for normal distribution, and the homogeneity of variance determined whether it was parametric or non‐parametric data. The statistical analysis was performed using GraphPad Prism (GraphPad Software). First, the data from Experiment 1–4 and migration assay using MCP1 were assessed using two‐way ANOVA. When an interaction between the additive reagents and HS treatment was not detected, each data set was assessed using Tukey–Kramer test. The data of migration assay using the culture supernatant were assessed using one‐way ANOVA. Moreover, Student's *t*‐test was conducted on the data for *CD80, IL6 Receptor, CCR2* mRNA expression, and Experiment 6. Probabilities less than 5% (*p *< .05) were considered statistically significant.

## 3 RESULTS

### 3.1 Experiment 1: Effect of HS and LPS on the cell viability in bovine endometrial epithelial and stromal cells

**Figure 1:**
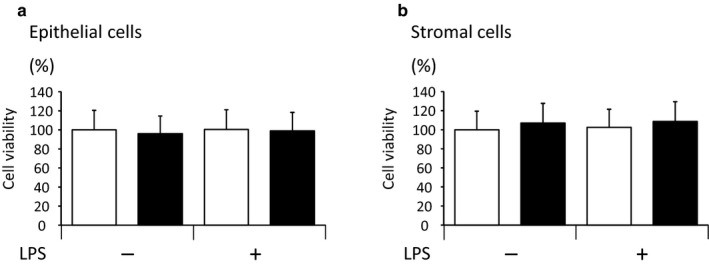
Effect of heat stress (HS) and lipopolysaccharide (LPS) on the cell viability of bovine endometrial epithelial (a) and stromal (b) cells (mean ± *SEM*, *n* = 8). Cell viability was determined as the relative portion of the endometrial cells in the absence of LPS at 38.5°C. The open and closed bars indicate the control and HS conditions, respectively

As shown in Figure 1, neither HS nor LPS affected the cell viability of the bovine endometrial epithelial and stromal cells (Figure 1a,b).

### 3.2 Experiment 2: Effect of HS and LPS on the mRNA expression of chemokines in cultured bovine endometrial epithelial and stromal cells

In Experiment 2, we investigated whether HS affects chemokine mRNA expression in bovine endometrial epithelial and stromal cells.

**Figure 2:**
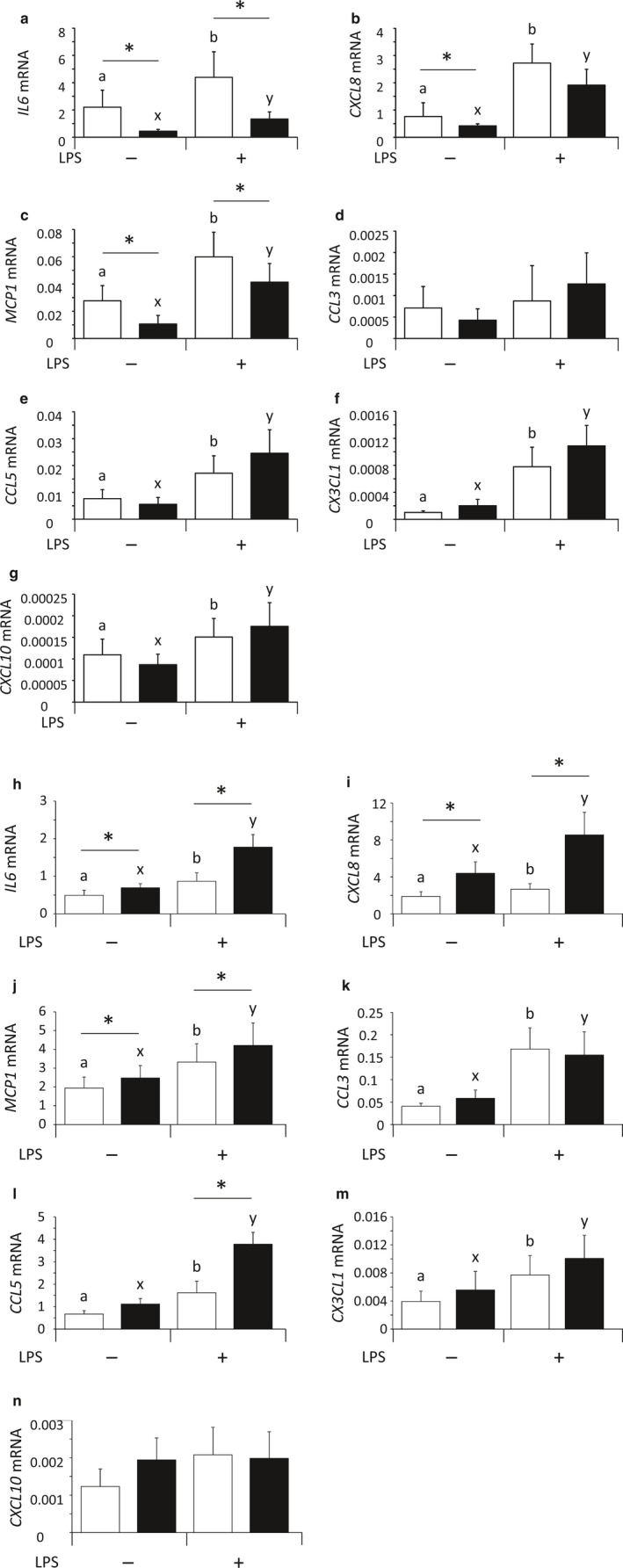
Effect of heat stress (HS) and lipopolysaccharide (LPS) on the mRNA expression of *IL6* (a, h), *CXCL8* (b, i), *MCP1* (c, j), *CCL3* (d, k), *CCL5* (e, l), *CX3CL1* (f, m), and *CXCL10* (g, n) in cultured bovine endometrial epithelial and stromal cells (mean ± *SEM*, *n* = 6–10). Each mRNA expression level was indicated as their copy number normalized by the expression of GAPDH. The open and closed bars indicate the control and HS conditions, respectively. a, b: Significant differences among groups cultured at 38.5°C (*p* < .05). x, y: Significant differences among groups cultured at 40.5°C (*p *< .05). *: Significant differences between 38.5°C and 40.5°C in the presence or absence of LPS (*p* < .05)

In endometrial epithelial cells, LPS significantly (*p *< .05) increased the mRNA expression of *IL6*, *MCP1, CXCL8*, *CCL5, CX3CL1,* and *CXCL10* (Figure 2a–c,e–g). Moreover, HS significantly (*p *< .05) suppressed the mRNA expression of *IL6* and *MCP1* regardless of the presence of LPS stimulation (Figure 2a,c). The mRNA expression of *CXCL8* was significantly (*p *< .05) suppressed under the HS conditions in the absence of LPS but HS did not affect the mRNA expression of *CXCL8* in the presence of LPS (Figure 2b). The mRNA expression of *CCL3*, *CCL5, CX3CL1,* and *CXCL10* was not influenced under HS conditions (Figure 2d–g). In addition, the mRNA expression of *CCL3*, *CCL5*, *CX3CL1,* and *CXCL10* was lower than the mRNA expression of *MCP1* (Figure 2c–g).

In contrast, in endometrial stromal cells, LPS significantly (*p *< .05) increased the mRNA expression of *IL6*, *MCP1, CXCL8*, *CCL3*, *CCL5,* and *CX3CL1* (Figure 2h–m). In the absence of LPS, HS significantly (*p *< .05) enhanced the mRNA expression of *IL6*, *MCP1,* and *CXCL8* (Figure 2h–j), while the mRNA expression of *IL6*, *MCP1*, *CXCL8,* and *CCL5* was enhanced by HS only in the presence of LPS (Figure 2h–j,l). The mRNA expression of *CCL3*, *CX3CL1,* and *CXCL10* was not affected by HS regardless of the presence or absence of LPS (Figure 2k,m,n).

In both types of endometrial cells, CXCL11 mRNA expression was undetectable in both the presence and absence of LPS (data not shown).

### 3.3 Experiment 3: Effect of HS and LPS on the production of IL‐6, MCP1, and IL‐8 in cultured bovine endometrial epithelial and stromal cells

**Figure 3:**
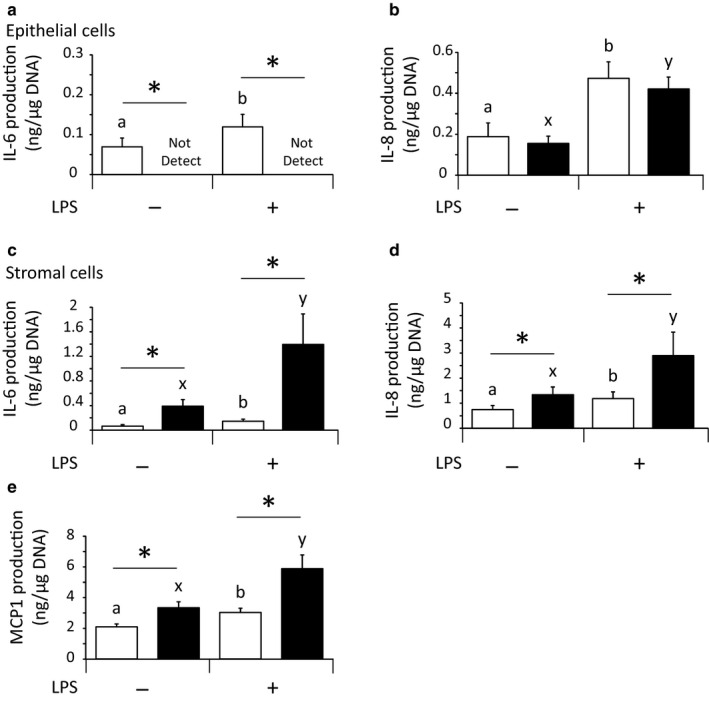
Effect of heat stress (HS) and lipopolysaccharide (LPS) on the production of IL‐6 (a, c), IL‐8 (b, d), and MCP1 (e) in cultured bovine endometrial epithelial and stromal cells (mean ± *SEM*, *n* = 8). The open and closed bars indicate the control and HS conditions, respectively. a, b: Significant differences among groups cultured at 38.5°C (*p *< .05). x, y: Significant differences among groups cultured at 40.5°C (*p *< .05). *: Significant differences between 38.5°C and 40.5°C in the absence or presence of LPS (*p *< .05)

As shown in Figure 3, we examined the effect of HS on chemokine secretion in bovine endometrial epithelial and stromal cells.

In the endometrial epithelial cells cultured at 38.5°C, the production of IL‐6 was significantly (*p *< .05) stimulated by LPS; however, it decreased to an undetectable level (<0.056 ng/ml) under HS conditions (Figure 3a). IL‐8 production by endometrial epithelial cells was significantly (*p *< .05) induced by LPS in both the control and HS conditions, then HS did not alter the effect of LPS on IL‐8 production (Figure 3b). MCP1 in endometrial epithelial cells was at an undetectable level in all treatment groups.

In bovine endometrial stromal cells, the production of IL‐6, MCP1, and IL‐8 was significantly (*p *< .05) increased by LPS. Moreover, HS significantly (*p *< .05) enhanced these increases in endometrial stromal cells (Figure 3c–e).

### 3.4 Experiment 4: Effect of HS and LPS on the mRNA expression of *TLR4*, *CD14,* and *MD2* in cultured bovine endometrial epithelial and stromal cells

In Experiment 4, to determine whether HS affects the recognition of LPS in bovine endometrial epithelial and stromal cells, we examined the mRNA expression of the pattern‐recognition receptor and its related factors.

**Figure 4:**
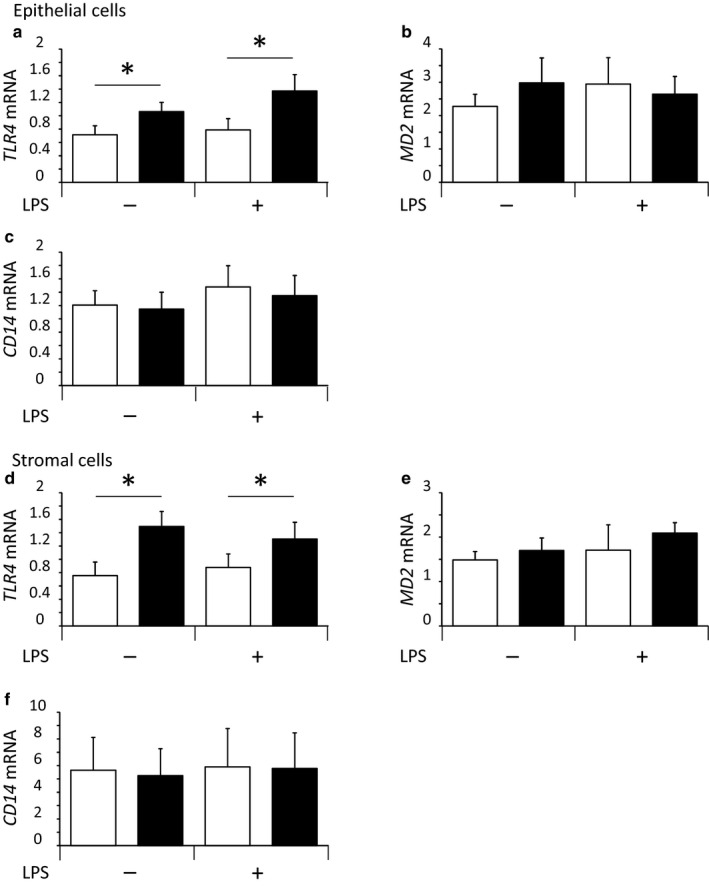
Effect of heat stress (HS) and lipopolysaccharide (LPS) on the mRNA expression of *TLR4* (a, d), *MD2* (b, e), and *CD14* (c, f) in cultured bovine endometrial epithelial and stromal cells (mean ± *SEM*, *n* = 10). Each mRNA expression level was indicated as their copy number normalized by the expression of GAPDH. The open and closed bars indicate the control and HS conditions, respectively. *: Significant differences between 38.5°C and 40.5°C in the absence or presence of LPS (*p *< .05)

As shown in Figure 4, while LPS did not affect the mRNA expression of *TLR4*, HS significantly (*p *< .05) increased it in endometrial epithelial and stromal cells (Figure 4a,d). Moreover, neither HS nor LPS affected the mRNA expression of *CD14* and *MD2* in both types of bovine endometrial cells (Figure 4b,c,e,f).

### 3.5 Experiment 5: Effect of HS on the mRNA expression of *IL6 Receptor* and *CCR2* in MΦs and their migration ability

In this experiment, the effect of HS on the migration ability of MΦs was investigated.

**Figure 5:**
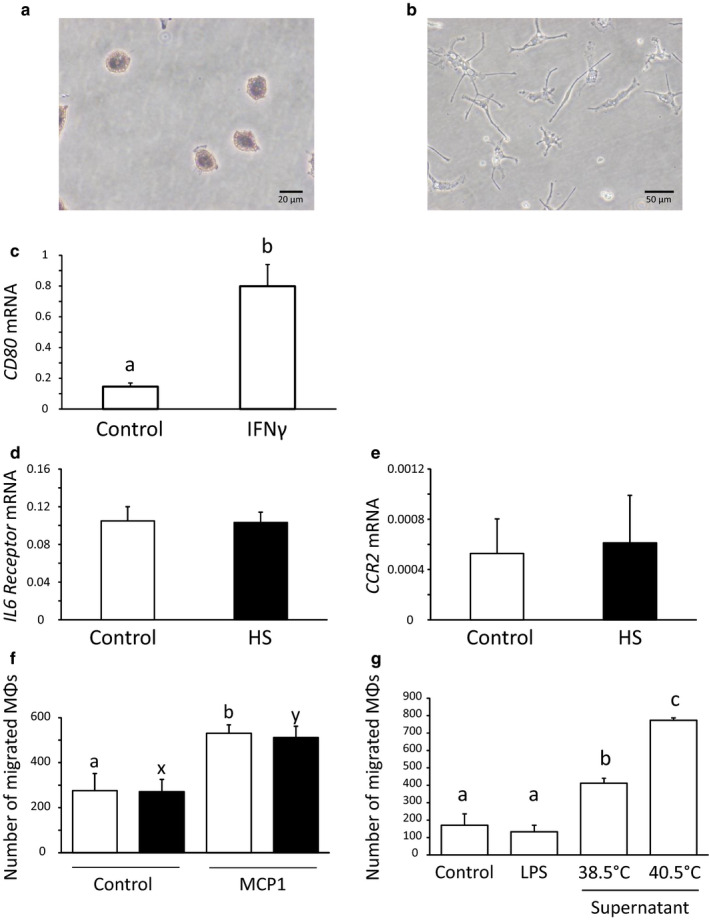
Evaluation of the isolation of monocytes (a) and differentiation of them into MΦs (b, c). Effect of HS on the mRNA expression of *IL6 Receptor* (d) and *CCR2* (e) in MΦs and migration ability of MΦs (f) (mean ± *SEM*, *n* = 5). The effect of culture supernatant of the endometrial stromal cells on the attraction of MΦs (g). The concentrated supernatant of endometrial stromal cells under control and HS conditions was indicated as 38.5°C and 40.5°C, respectively (d). Each mRNA expression level was indicated as their copy number normalized by the expression of GAPDH. The open and closed bars indicate the control and HS conditions, respectively. a, b: Significant differences among groups cultured at 38.5°C (*p *< .05). x, y: Significant differences among groups cultured at 40.5°C (*p *< .05)

After the specific and non‐specific esterase staining, we confirmed that more than 80% of the attached cells in the culture flasks were monocytes (Figure 5a). In addition, as shown in Figure 5, IFNγ‐stimulated monocytes appeared pseudopodia and increased the *CD80* mRNA expression (Figure 5b,c).

As shown in Figure 5, HS did not affect the mRNA expression of *IL6 Receptor* and *CCR2* in the MΦs (Figure 5d,e). MCP1 significantly (*p *< .05) attracted MΦs under both the control and HS conditions; however, the migration ability of the MΦs was not affected by HS (Figure 5f). In addition, the culture supernatant of the endometrial stromal cells under the HS condition significantly (*p *< .05) attracted MΦs compared with their supernatant under the control condition. Moreover, LPS did not affect the migration ability of the MΦs (Figure 5g).

### 3.6 Experiment 6: Localization of MΦs in bovine endometrium in summer and winter

**Figure 6:**
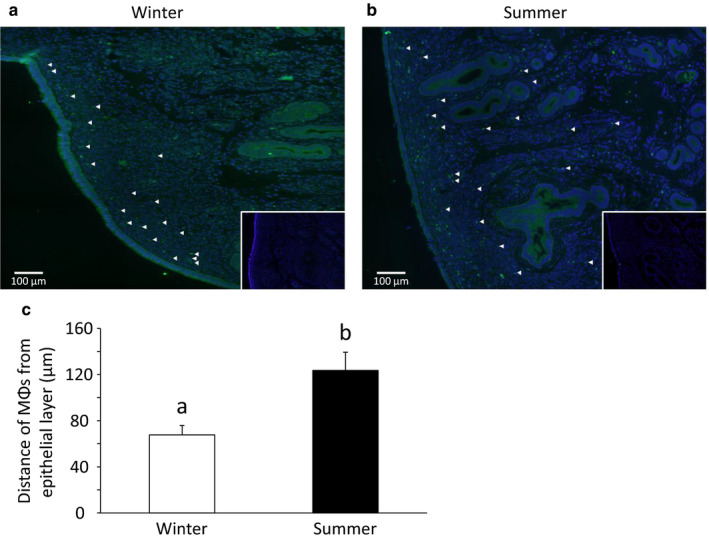
The localization of MΦs in bovine endometrium in summer and winter. Arrow heads indicate the MΦs (a, b). The distance from the epithelial layer to MΦs (c) (mean ± *SEM*, *n* = 6). Different superscripts indicate a significant difference (*p *< .05)

MΦs were sparsely localized through the bovine endometrium in the summer whereas they were localized in the subepithelial stroma in the winter (Figure 6a,b). The distance of the MΦs from the epithelial layer was significantly (*p *< .05) longer in the summer than in the winter (Figure 6c).

## 4 DISCUSSION

One type of bacteria that cause endometritis is *E. coli,* and it localizes in the uterine lumen and beneath the epithelial layer in the infected uterus (Karstrup et al., 2017). To eliminate these bacteria, immune cells need to be recruited to the epithelial layer (Karstrup et al., 2017) and neutrophils play an important role in the elimination of bacteria, especially (Brodzki et al., 2014; Sheldon et al., 2014). Neutrophils in peripheral blood recognize the gradient of IL‐8 concentrations and are chemoattracted to the infection site (Martínez‐Burgo et al., 2019; Zachariae, 1993). In the present study, HS enhanced IL‐8 production in bovine endometrial stromal cells (Figure 3d) but did not affect it in endometrial epithelial cells (Figure 3b). Because IL‐8 production in endometrial epithelial cells, a major site for the recognition of the LPS produced by *E. coli*, was not decreased by HS, the recruitment of neutrophils might not be influenced by HS.

Following the recruitment of neutrophils, MΦs are attracted by some chemokines secreted by endometrial cells. These attracted MΦs contribute to the elimination of bacteria (Brodzki et al., 2014), the orchestration of inflammation (Dadarwal et al., 2017), the activation of T‐cells and B‐cells (Fischer et al., 2010; Kauma, 2000), and tissue repair (Sheldon et al., 2014). In the current study, HS enhanced the MCP1 production induced by LPS in endometrial stromal cells (Figure 3e). However, in endometrial epithelial cells, MCP1 production was not detected under either the control or HS conditions regardless of the presence of LPS. Therefore, in the endometrial epithelial cells, MCP1 might not contribute to the recruitment of MΦs to the endometrial subepithelial stroma. Moreover, HS enhanced the IL‐6 production in the endometrial stromal cells (Figure 3c) but suppressed it in the endometrial epithelial cells (Figure 3a). In addition, HS enhanced *CCL5* mRNA expression induced by LPS but did not affect the mRNA expression of *CCL3*, *CX3CL1,* and *CXCL10* in the endometrial stromal cells (Figure 3k–n). Moreover, the supernatant of endometrial stromal cells cultured under HS conditions attracted a large number of MΦs (Figure 5g). Taken together HS might affect the localization of MΦs in the endometrium. Indeed, MΦs were mainly localized subepithelial stroma in winter, whereas they were sparsely localized throughout the endometrium in summer (Figure 6a–c). However, it is still unclear whether this change in MΦ localization under HS condition causes the prolongation of endometritis symptoms in summer. Therefore, further in vivo study is necessary.

Interestingly, we found that HS had opposite effects on chemokine production between endometrial epithelial and stromal cells. We presumed that difference of bacterial sensing by its specific receptor was involved in this opposite reaction in both types of endometrial cells. However, HS did not affect the mechanism for the recognition of LPS because HS upregulated only TLR4 but did not MD‐2 and CD14 in bovine endometrial epithelial and stromal cells (Figure 4a–f). These results suggest that there is respective different mechanism(s) for the opposite reaction of chemokine production in endometrial epithelial and stromal cells under HS condition. Indeed, similar phenomena are observed in several types of neighboring cells. In the bovine endometrial epithelial and stromal cells, *Trueperella pyogenes* induces cytolysis of endometrial stromal cells but not of endometrial epithelial cells, because endometrial stromal cells contain more cholesterol than endometrial epithelial cells (Amos et al., 2014). In addition, under the ischemic and hypoxic stress conditions, the neuronal cells undergo apoptosis but astrocytes have tolerated, nevertheless, they are neighboring cells, because astrocytes have unique endoplasmic reticulum stress transducers (Kondo et al., 2005). Therefore, to get the clue for the improvement of the local effect of HS on chemokine production, further studies on the cellular mechanism(s) in endometrial epithelial and stromal cells, individually, are necessary.

## 5 CONCLUSION

**Figure 7:**
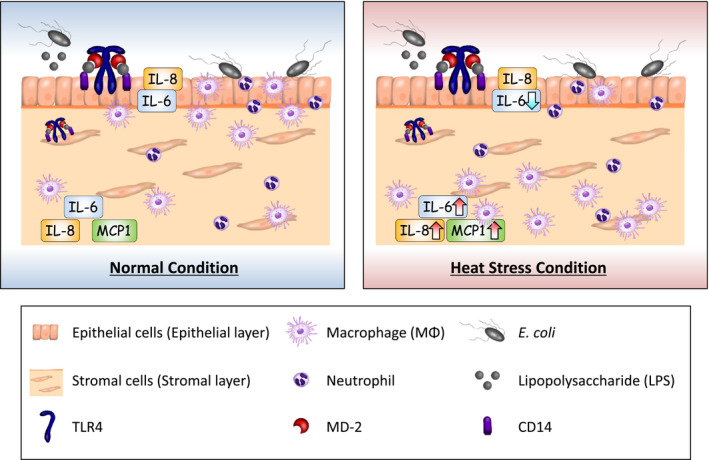
Illustration depicting our conclusion. Heat stress (HS) increased IL‐6, MCP1, and IL‐8 production in endometrial stromal cells but suppressed IL‐6 production in endometrial epithelial cells. This disruption of chemokine production in both types of endometrial cells might cause the alteration of MΦs localization in the endometrium under heat stress (HS) conditions

Our present results suggested that HS caused alteration of the chemokine production in bovine endometrial epithelial and stromal cells with the respective different mechanisms. This change in chemokine production might affect the recruitment of MΦs and alter their localization in the endometrium in summer (Figure 7).

## 6 PERSPECTIVE

The present in vitro study provided valuable information about the effect of HS on immune response in bovine endometrium at the cellular level. In contrast, HS affects several systemic physiological functions in cattle; for example, voluntary reduction of feed intake, panting, and hypothalamic‐pituitary‐adrenal (HPA) axis (Bagath et al., 2019; Hansen, 2020; Sanchez et al., 1994). HS stimulates the HPA axis and enhances the production of cortisol, resulting in the suppression of the immune system (Grandin, 1997). These systemic effects of HS are closely related to the immune responses at the local (cellular) level. Therefore, to confirm the idea found in the present in vitro study, further in vivo and in vitro studies should be necessary.

## CONFLICT OF INTEREST

None of the authors has any potential conflicts of interest associated with this research.

## AUTHORS’ CONTRIBUTIONS

S.S. planned the experimental design, collected all data, and wrote the first manuscript. T.H. and Y.Y. planned a portion of the experimental design. K.K. developed the original experimental design. S.S. and K.K. reviewed and edited the manuscript. All authors have read and approved the manuscript.
